# Adrenocorticotropic hormone combined with magnesium sulfate therapy for infantile epileptic spasms syndrome: a real-world study

**DOI:** 10.1007/s12519-023-00771-1

**Published:** 2023-12-09

**Authors:** Wen He, Qiu-Hong Wang, Jiu-Wei Li, Yang-Yang Wang, Xiao-Mei Luo, Lin Wan, Jing Wang, Xiu-Yu Shi, Wei-Hua Zhang, Fang Fang, Li-Ping Zou

**Affiliations:** 1 Senior Department of Pediatrics, the Seventh Medical Center of Chinese PLA General Hospital, Department of Pediatrics, the First Medical Center of Chinese PLA General Hospital, Beijing 100853, China; 2 Department of Neurology, Beijing Children’s Hospital, Capital Medical University, Beijing, China; 3 Center of Epilepsy, Beijing Institute for Brain Disorders, Beijing 100069, China

**Keywords:** Adrenocorticotropic hormone, Clinical trial, Infantile epileptic spasms syndrome, Magnesium sulfate, Real-world study

## Abstract

**Background:**

Infantile epileptic spasms syndrome (IESS) is a serious disease in infants, and it usually evolves to other epilepsy types or syndromes, especially refractory or super-refractory focal epilepsies. Although adrenocorticotropic hormone (ACTH) is one of the first-line and effective treatment plans for IESS, it has serious side effects and is not sufficiently effective.

**Methods:**

A retrospective study of the clinical outcomes of ACTH combined with magnesium sulfate (MgSO_4_) therapy for IESS in two hospital centers was conducted. The major outcome of the single and combined treatment was evaluated by changes in seizure frequency and improvements in hypsarrhythmia electroencephalography (EEG). To reduce the confounding bias between the two groups, we used SPSS for the propensity score matching (PSM) analysis.

**Results:**

We initially recruited 1205 IESS patients from two Chinese hospitals and treated them with ACTH combined with MgSO_4_ and ACTH alone. Only 1005 patients were enrolled in the treatment (ACTH combined with MgSO_4_: 744, ACTH: 261), and both treatment plans had a more than 55% response rate. However, compared to patients treated with ACTH alone, those patients treated with ACTH combined with MgSO_4_ had better performance in terms of the seizure frequency and hypsarrhythmia EEG. After PSM, the two groups also showed significant differences in responder rate [70.8% (95% confidence interval, CI) = 66.7%–74.8%) vs. 53.8% (95% CI = 47.4%–60.2%), *P* < 0.001], seizure frequency (*P* < 0.001) and hypsarrhythmia EEG resolution (*P* < 0.001). Notably, multivariate analysis revealed that the lead time to treatment and the number of antiseizure medications taken before treatment were two factors that may affect the clinical outcome. Patients with less than 3 months of lead time responded to the treatment much better than those with > 3 months (*P* < 0.05). In addition, the overall incidence of adverse reactions in the ACTH combined with MgSO_4_ group was much lower than that in the ACTH group (31.4% vs. 63.1%, *P* < 0.001). During the treatment, only infection (*P* = 0.045) and hypertension (*P* = 0.025) were significantly different between the two groups, and no baby died.

**Conclusion:**

Our findings support that ACTH combined with MgSO_4_ is a more effective short-term treatment protocol for patients with IESS than ACTH alone, especially for those patients with short lead times to treatment.

Video Abstract (MP4 533623 KB)

**Supplementary Information:**

The online version contains supplementary material available at 10.1007/s12519-023-00771-1.

## Introduction

Infantile epileptic spasms syndrome (IESS), also known as infantile spasms, is a serious disease for infants with a low incidence rate (0.25–0.42/1000 live births) [1]. It frequently evolves to other epilepsy types or syndromes, especially refractory or super-refractory focal epilepsies. The electroencephalography (EEG) of IESS patients displays hypsarrhythmia, which can be characterized by a widespread and chaotic mixed waveform composed of high amplitude slow and spike waves in various brain regions. In 2007, some scholars proposed that hypsarrhythmia EEG is an epileptic electrical status and a form of non-convulsive status in infants [2, 3]. In 2012, the expert group of American non-convulsive status epilepticus (NCSE) included hypsarrhythmia EEG for IESS in the definition of the EEG standard of NCSE [4]. The International League Against Epilepsy (ILAE) also considers IESS as an important trigger for both convulsive and NCSE in infants [5]. However, the effect of anticonvulsant status epileptic drugs on the resolution of hypsarrhythmia EEG in IESS patients is still unknown.

Since 1901, there have been sporadic cases of magnesium infusion in the treatment of seizures. In recent years, magnesium sulfate (MgSO_4_) has been widely used in the prevention and control of seizures. Many studies have shown that magnesium is closely related to epilepsy. For example, an extracellular low magnesium environment may induce seizures [6–9]. The serum magnesium concentration of epileptic patients is lower than normal, and magnesium supplementation can help control seizures [10–14]. In previous animal experiments, magnesium blocked N-methyl-D-aspartate (NMDA) receptors and reduced the activity of NMDA receptors by binding to calcium channels coupled with NMDA receptors, thus preventing calcium influx to play an antiepileptic role [15–18]. In addition, magnesium can inhibit the reuptake of glutamic acid and enhance the γ-aminobutyric acid receptor [19]. Thus, in 2012, MgSO_4_ was recommended as a second-line drug for the treatment of refractory and super-refractory convulsive status epilepticus by the consensus of American experts [20].

A real-world study refers to the research process of collecting data related to the health of research subjects (real-world data) or summarizing data derived from these data in a real-world environment for predetermined clinical problems. In 2006, our team registered a clinical study on the efficacy of adrenocorticotropic hormone (ACTH) combined with MgSO_4_ and ACTH alone in the International Society for Research on Cadmium and Trace Elements Toxicity (ISRCTN786541ll). We found that compared with ACTH treatment, the combination treatment (ACTH combined with MgSO_4_) had advantages in relieving seizures, maintaining normal EEG, and improving individual social neural development for IESS patients, as well as a lower incidence of adverse events and better tolerance [21]. In 2012, the American Academy of Neurology and the Child Neurology Society recommended ACTH, prednisone and vigabatrin as the first-line standard treatment plan for IESS patients, but they also pointed out that the combination treatment plan may be the future [22]. However, the limited sample size and short follow-up time make it difficult to obtain sufficient information on the safety of treatment plans and hard to extrapolate trial conclusions in real clinical environments. It is emerging to expand the study with more participants in the real world.

In the present study, we analyzed the data from a large sample size of IESS patients accumulated over these years to conduct a more comprehensive evaluation of the effectiveness, safety, and other aspects of ACTH combined with MgSO_4_ therapy in real-world medical practice.

## Methods

### Patients

This was a retrospective study. We enrolled patients diagnosed with IESS who were admitted to the General Hospital of PLA and Beijing Children’s Hospital of Capital Medical University between May 2013 and October 2018 as our study subjects. The discharge diagnosis of “infantile spasms syndrome” was identified through a comprehensive search in the hospital information system. Simultaneously, individuals with incomplete information records, repeated hospitalizations, and those meeting the exclusion criteria were excluded from the analysis. The inclusion criteria were as follows: (1) two months to two years old; (2) diagnosed with IESS by a child neurologist; (3) standard, sleep, long range, and video EEGs interpreted by neurophysiologists, presenting diffuse high amplitude slow rhythms, extensive spike slow activity, and localized, multifocal spikes and sharp waves with typical (or atypical) hypsarrhythmia; (4) receiving either ACTH alone or ACTH combined with MgSO_4_ as a treatment for IESS; and (5) the antiseizure medications (ASMs) were not adjusted within two weeks, and the frequency of epileptic seizures remained stable. The exclusion criteria were as follows: (1) other serious diseases (e.g., acute and infectious diseases) that are not suitable for this study, such as septicemia, pneumonia, urinary tract infection, and electrolyte disorders; (2) diagnosed with pyridoxine-dependent epilepsy; (3) taking prednisone orally; and (4) participating in other medical experiments. The parents or guardians of all enrolled patients signed an informed consent form, and the research plan was approved by the Ethics Committee of Chinese PLA General Hospital (No. 2023–120).

### Study design and treatment strategy

In this retrospective study, we divided the enrolled children into two groups according to the treatment method. Treatment group: patients received combined treatment with ACTH (2–2.5 U/kg/day, Shanghai No. 1 Biomedical & Pharmaceutical Co., Ltd, Shanghai, China) and MgSO_4_ (0.25 g/kg/day, Tianjin Jinyao Pharmaceutical Co., Ltd., Tianjin, China). Control group: patients only received ACTH (2–2.5 U/kg/day). All the patients from the participating hospitals used the same products. Every day, patients received intravenous infusion with ACTH combined with MgSO_4_ or ACTH dissolved in 100 mL 5% dextrose at a constant rate for at least 6 hours. During the medication, the patients were closely guarded by experienced pediatricians and nurses, and their blood pressure and other vital signs were regularly monitored. The treatment was continued for two weeks and could be discriminated when there were adverse reactions such as infection, hypertension, hypokalemia, and other events that patients could not tolerate. An EEG examination was performed for the patients before and after the treatment by neurophysiologists.

The major outcome of the single and combined treatment was evaluated by changes in seizure frequency and improvements in hypsarrhythmia EEG. The responders were defined as individuals who achieved a reduction in seizure frequency of ≥ 50% compared to before treatment and the resolution of hypsarrhythmia EEGs. Non-responders were defined as individuals who had less than a 50% reduction in seizure frequency compared to before treatment, or although they may have achieved a reduction of ≥ 50% in seizure frequency but without improvement observed in their hypsarrhythmia EEGs. The minor outcomes include the response time, which was defined as the time from the treatment start time to the cessation of epileptic seizures, and the rate of resolution of hypsarrhythmia EEG, which was defined as complete disappearance of hypsarrhythmia EEG during the waking and sleeping periods after two weeks of treatment. The presence of atypical hypsarrhythmia or persistently visible hypsarrhythmia waveforms is defined as unresolved hypsarrhythmia EEG.

### Data collection

The retrospective data were collected from the hospital information systems of the two hospitals and inputted into the Epidata database by four pediatric neurology experts who have received training on the Epidata database. Two specialists in quality control conducted a thorough review and cleaning of the data. The standard for data entry quality control required an error rate of less than 0.1% to minimize any potential bias. Demographic characteristics were collected for all enrolled patients, and the clinical characteristics were evaluated, including (1) disease history (e.g., birth, family, perinatal and neonatal brain injury history including neonatal hypoxic ischemic encephalopathy, neonatal hypoglycemic encephalopathy, intracranial hemorrhage, and encephalitis), and the epileptic seizure types were classified according to the ILAE 2017 guidelines [23]; (2) physical examination and professional examination of the nervous system to determine whether there were neurocutaneous syndromes (e.g., nodular sclerosis, craniofacial angiomatosis, Menkes disease), Down’s syndrome, and other special facial and developmental abnormalities; (3) imaging examination of brain scan including computerized X-ray tomography (CT), positron emission tomography, and magnetic resonance imaging (MRI); and (4) metabolic screening (urine and blood) for phenylketonuria, methylmalonic acidemia and other metabolic disorders.

### Gene testing

Gene testing was performed after obtaining the informed consent of the guardian of each patient. Veinous blood was obtained from the patient and sequenced at the Beijing Institute of Genomics with one or more of the following protocols: the epilepsy gene panel, whole-exome sequencing, Sanger sequencing and multiplex ligation-dependent probe amplification. Based on the sequencing results and clinical interpretation needs, it was decided whether to perform Sanger sequencing to verify the high-throughput sequencing results. The pathogenicity of gene mutations was comprehensively evaluated based on the latest American College of Medical Genetics and Genomics grading criteria and the clinical manifestations of children.

### Etiological classification

The etiologies for IESS are divided into three main etiological categories according to the 2010 guidelines of the International Anti-Epilepsy Alliance: structural/metabolic, genetic, and unknown etiologies [24]. The etiologies of IESS patients in this study were classified by professional clinicians and genetics consultants based on the clinical history, laboratory tests, and metabolic screening (blood and urine). According to the ILAE 2010 guidelines, tuberous sclerosis complex is classified as the etiology of structures.

### Propensity score matching

To reduce the confounding bias between the two groups, we used SPSS (v22) for propensity score matching (PSM) analysis. To maximize the utilization of these real-world data, the ratio of control and treatment groups was set as 1:3. The caliper matching method was used for PSM, and the cutoff for caliper was 0.02. Confounders used for PSM included gender, age of diagnosis, the number of ASMs taken before enrollment, family history of nervous system disease (NSD), etiology classification, and the presence of diseases that affect the development of the nervous system in the neonatal period.

### Statistical analyses

Descriptive analyses were conducted on the baseline information of patients, including gender, age, and age of diagnosis. To compare the frequency of seizures, all seizure frequencies were normalized based on the number of episodes within a day, rather than the number of series of episodes, and only the total frequency of episodes within a day was calculated. The difference in continuous variables between the two groups was measured using independent *t* tests and Mann‒Whitney *U* tests. The difference in categorical variables between the two groups was calculated using Pearson’s Chi-square test and Fisher’s exact test. A logistic regression model was constructed to analyze the possible factors that may increase or decrease the treatment efficacy, and the significance *P* value was set as 0.05 (two-tailed). All statistical analyses were conducted using SPSS (v22).

### Definitions of keywords

Lead time: the time interval between the onset of an epileptic spasm and enrollment for treatment; refractory IESS: failed in the treatment of two or more ASMs before enrollment in this study.

## Results

### Clinical characteristics of enrolled patients

**Fig. 1:**
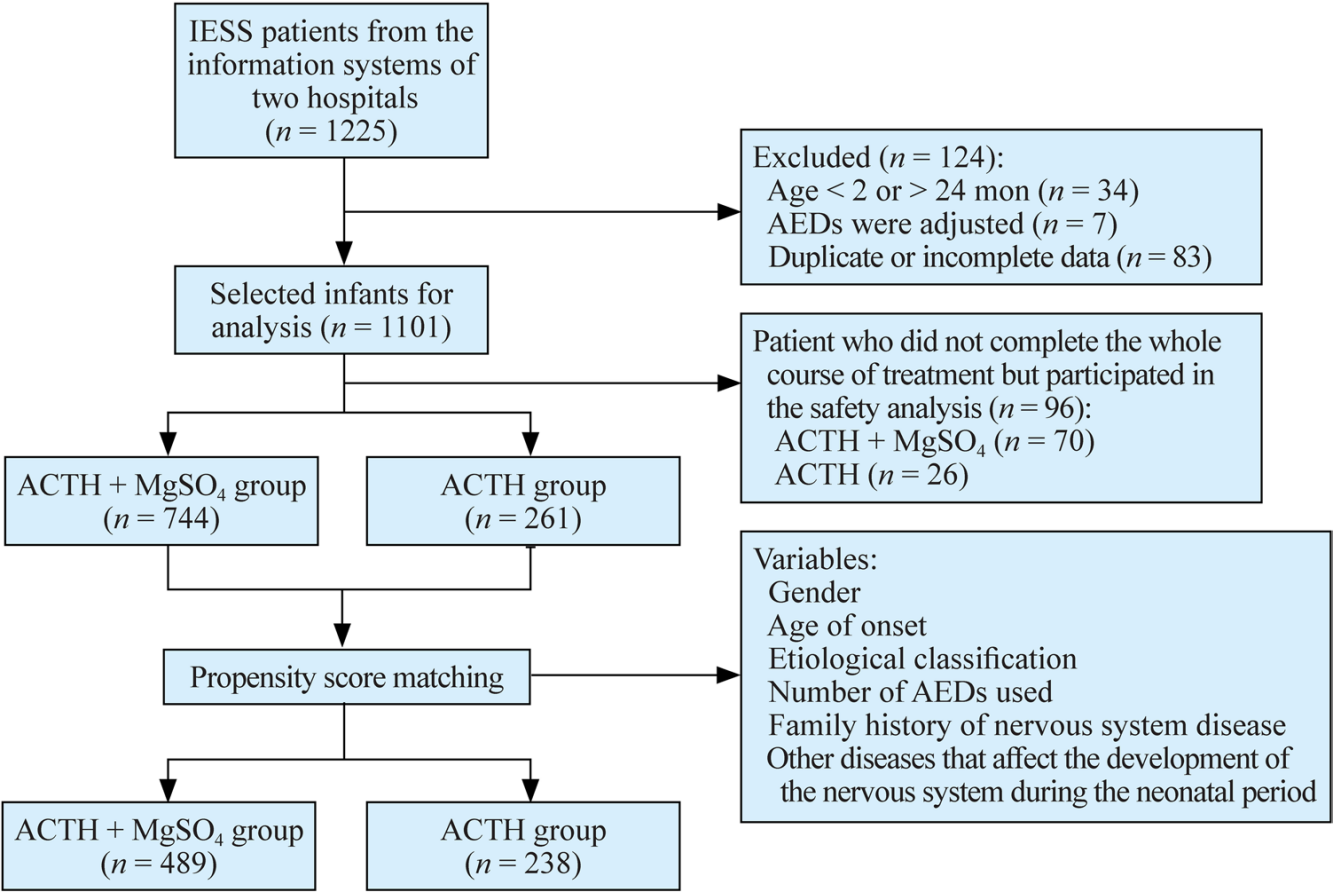
Flowchart of patient selection process. *IESS* infantile epileptic spasms syndrome, *AED* anti-epileptic drug, *ACTH* adrenocorticotropic hormone, *MgSO*_*4*_ magnesium sulfate

To explore the treatment effect of ACTH combined with MgSO_4_ for IESS patients, we conducted this retrospective study, as described in Fig. 1**.** A total of 1225 patients were extracted from the information systems of the two hospitals. After rigorous screening, patients who did not meet the inclusion criteria were excluded, along with those having duplicate hospitalization information (only including data from their first treatment) and patients with incomplete information. Ultimately, a total of 1101 individuals diagnosed with IESS were included in this study. They were categorized into two groups based on the treatment regimen administered during hospitalization: the ACTH combined with MgSO_4_ group (*n* = 814) and the ACTH monotherapy group (*n* = 287). While 96 patients did not finish the fortnight treatment, we finally obtained 744 patients receiving ACTH combined with MgSO_4_ treatment and 261 patients receiving ACTH treatment only. The clinical characteristics of these patients are shown in Table 1. The mean age of IESS patients was 10 months, and the age of onset was younger than 6 months. More than 90% of the enrolled patients were full-term infants, had flexor spasms and had no family history of NSD. We also noticed that more than half of the patients had taken more than two kinds of ASMs. Interestingly, most of the IESS patients had no epilepsy before the diagnosis of IESS and had no other diseases that may affect the nervous system, and the etiological classification of 54.4% of patients was unknown. It is notable that before PSM, the two groups had significant differences in some kinds of characteristics, such as sex, age of onset, number of ASMs used, family history of NSD, etiological classification and disease that affects the nervous system during the neonatal period. After PSM, the difference in all clinical characteristics disappeared, and 489 and 238 IESS patients received the combination treatment of ACTH combined with MgSO_4_ and ACTH, respectively.

**Table 1:** Clinical characteristics of enrolled IESS patients before and after PSM

Covariates	All patients			After PSM		
	ACTH combined with MgSO_4_ (*n* = 744)	ACTH (*n* = 261)	*P*	ACTH combined with MgSO_4_ (*n* = 489)	ACTH (*n* = 238)	*P*
Gender, *n* (%)			0.050			0.687
Male	445 (59.8)	174 (66.7)		311 (63.6)	155 (65.1)	
Female	299 (40.2)	87 (33.3)		178 (36.4)	83 (34.9)	
Age (mon)	10.0 (2.1–23.8)	10.0 (2.7–23.9)	0.843	9.6 (2.1–23.8)	10.0 (2.7–23.9)	0.218
Age of onset (mon)	4.9 (0.1–19.0)	5.4 (0.1–19.0)	0.044	5.0 (1.0–19.0)	5.2 (0.1–15.0)	0.438
Lead time to treatment (mon)	5.1 (0.1–22.7)	4.7 (0.3–20.7)	0.214	4.6 (0.1–21.2)	4.8 (0.3–20.7)	0.402
Types of spasms, *n* (%)			0.238			0.512
Flexor	688 (92.6)	248 (95.0)		458 (93.7)	226 (95)	
Extensor	35 (4.7)	6 (2.3)		18 (3.7)	5 (2.1)	
Flexor–extensor	20 (2.7)	7 (2.7)		13 (2.7)	7 (2.9)	
Number of ASMs taken	2.2 (0.0–7.0)	1.7 (0.0–7.0)	< 0.001	2.0 (0.0–7.0)	1.8 (0.0–7.0)	0.081
< 2	247 (33.2)	126 (48.3)		196 (40.1)	107 (45.0)	
≥ 2	497 (66.8)	135 (51.7)		293 (59.9)	131 (55.0)	
Gestational age, *n* (%)			0.916			0.756
Full-term	676 (90.9)	237 (90.8)		445 (91.0)	215 (90.3)	
Premature	64 (8.6)	22 (8.4)		42 (8.6)	21 (8.8)	
Post-term	4 (0.5)	2 (0.8)		2 (0.4)	2 (0.8)	
Family history of NSD, *n* (%)			0.025			0.879
Yes	13 (1.7)	11 (4.2)		9 (1.8)	4 (1.7)	
No	731 (98.3)	250 (95.8)		480 (98.2)	234 (98.3)	
Epilepsy before diagnosis, *n* (%)			0.181			0.644
Yes	160 (21.5)	46 (17.6)		89 (18.2)	40 (16.8)	
No	584 (78.5)	215 (82.4)		400 (81.8)	198 (83.2)	
Etiological classification, *n* (%)			< 0.001			0.136
Known pathogeny	377 (50.7)	81 (31.0)		188 (38.4)	78 (32.8)	
Structure/Metabolism	307 (41.3)	79 (30.3)		162 (33.1)	76 (31.9)	
Genetics	70 (9.4)	2 (0.8)		26 (5.3)	2 (0.8)	
Unknown pathogeny	367 (49.3)	180 (69.0)		301 (61.6)	160 (67.2)	
Disease that affects the nervous system during the neonatal period, *n* (%)			0.017			0.180
Yes	232 (31.2)	61 (23.4)		138 (28.2)	56 (23.5)	
No	512 (68.8)	200 (76.6)		351 (71.8)	182 (76.5)	

*IESS* infantile epileptic spasms syndrome, *PSM* propensity score matching, *ACTH* adrenocorticotropic hormone, *MgSO*_*4*_ magnesium sulfate, *ASMs* antiseizure medications, *NSD* nervous system disease

### Evaluation of the treatment efficacy of ACTH combined with MgSO_4_ and ACTH for IESS patients

Next, we evaluated the treatment effect of ACTH combined with MgSO_4_ and ACTH for IESS patients. In total, 744 IESS patients from the ACTH combined with MgSO_4_ group and 261 IESS patients from the ACTH group were enrolled in this study. As shown in Table 2, before treatment, the average seizure frequencies in the ACTH combined with MgSO_4_ and ACTH groups were 63.1 and 58.8 times per day, respectively, and there was no difference (*P* = 0.477). After two weeks of treatment, the average seizure frequency dropped to 10.8 and 25.5 times/day in the ACTH combined with MgSO_4_ and ACTH groups (*P* < 0.001), respectively. After treatment, 509 IESS patients from the ACTH combined with MgSO_4_ group and 144 IESS patients from the ACTH group achieved a reduction in seizure frequency of ≥ 50% compared to baseline and the resolution of hypsarrhythmia EEGs [68.4% (509/744), 95% confidence interval (CI) = 65.1%–71.8% vs. 55.2% (144/261), 95% CI = 49.1%–61.2%; *P* < 0.001]. Notably, complete response to treatment and successful cessation of seizures were observed in 444 IESS patients from the ACTH combined with MgSO_4_ group and 108 IESS patients from the ACTH group [59.7% (444/744) vs. 41.4% (108/261), *P* < 0.001]. No statistically significant difference was observed in the time between treatment initiation and seizure cessation, which was 7 and 8 days in the ACTH combined with MgSO_4_ and ACTH groups, respectively (*P* = 0.566) (Table 2). The mean baseline serum magnesium ion concentration in children in the ACTH combined with MgSO_4_ group was 0.90 ± 0.06 mmol/L, and the mean serum magnesium ion concentration after treatment was 1.02 ± 0.13 mmol/L. The difference in serum magnesium concentration before and after treatment was 0.11 ± 0.13 mmol/L, with a statistically significant difference (*t* = 23.33, *P* < 0.001, 95% CI = 0.10–0.12).

**Table 2:** Evaluation of the effect of ACTH combined with MgSO_4_ and ACTH treatment for IESS patients

Variables	All patients (*n* = 1005)			After PSM (*n* = 727)		
	ACTH combined with MgSO_4_ (*n* = 744)	ACTH (*n* = 261)	*P*	ACTH combined with MgSO_4_ (*n* = 489)	ACTH (*n* = 238)	*P*
Seizure frequency baseline (times/d)	63.1 (1–900)	58.8 (1–640)	0.477	60.2 (1–900)	59.1 (1–640)	0.867
Seizure frequency after treatment (times/d)	10.8 (0–285)	25.5 (0–455)	< 0.001	10.1 (0–260)	26.2 (0–455)	< 0.001
Disappeared hypsarrhythmia EEGs, *n* (%)			< 0.001			< 0.001
Yes	567 (76.2)	157 (60.2)		381 (77.9)	141 (59.2)	
No	177 (23.8)	104 (39.8)		108 (22.1)	97 (40.8)	
Responders, *n* (%)			< 0.001			< 0.001
Yes	509 (68.4)	144 (55.2)		348 (70.8)	128 (53.8)	
Complete	444 (59.7)	108 (41.4)		305 (62.4)	95 (39.9)	
No	235 (31.6)	117 (44.8)		143 (29.2)	110 (46.2)	
Responders in patients with ASMs ≥ 2 types, *n* (%)			0.002			0.002
Yes	318 (64.0)	67 (49.6)		191 (65.2)	64 (48.9)	
No	179 (36.0)	68 (50.4)		102 (34.8)	67 (51.1)	
Days the episode ends (d)	7	8	0.566	7	8	0.377

*ACTH* adrenocorticotropic hormone, *MgSO*_*4*_ magnesium sulfate, *ASMs* antiseizure medications, *EEG* electroencephalography

After PSM, the responders in the ACTH combined with MgSO_4_ and ACTH groups also had significant differences. After treatment, 348 IESS patients from the ACTH combined with MgSO_4_ group and 128 IESS patients from the ACTH group achieved a reduction in seizure frequency of ≥ 50% compared to baseline and the resolution of hypsarrhythmia EEGs [70.8% (348/489), 95% CI = 66.7%–74.8% vs. 53.8% (128/238), 95% CI = 47.4%–60.2%; *P* < 0.001]. In addition, 381 IESS patients in the ACTH combined with MgSO_4_ group and 141 IESS patients in the ACTH group had hypsarrhythmia EEGs that disappeared [77.9% (381/489) vs. 59.2% (141/238), *P* < 0.001]. The average seizure frequency dropped from 60.2 times/day to 10.1 times/day in the ACTH combined with MgSO_4_ groups compared to 59.1 to 26.2 times/day in the ACTH group (*P* < 0.001). Similarly, complete response to treatment and successful cessation of seizures were observed in 305 IESS patients from the ACTH combined with MgSO_4_ group and 95 IESS patients from the ACTH group [62.4% (305/489) vs. 39.9% (95/238), *P* < 0.001]. The average baseline serum magnesium ion concentration in children was 0.91 ± 0.06 mmol/L, and the average serum magnesium ion concentration after treatment was 1.01 ± 0.12 mmol/L. The difference in serum magnesium concentration before and after treatment was 0.11 ± 0.13 mmol/L, and the difference was still statistically significant (*t* = 18.08, *P* < 0.001, 95% CI = 0.09–0.11). This result indicated that the concentration of magnesium ions in the body of IS children significantly increased after treatment with ACTH combined with MgSO_4_. Interestingly, even in children with refractory IESS, ACTH combined with MgSO_4_ treatment demonstrated more significant therapeutic efficacy. After treatment, 191 refractory IESS patients from the ACTH combined with MgSO_4_ group and 64 refractory IESS patients from the ACTH group achieved a reduction in seizure frequency of ≥ 50% compared to baseline and the resolution of hypsarrhythmia EEGs [65.2% (191/293) vs. 48.9% (64/131), *P* = 0.002] (Table 2).

### Multivariate analysis for ACTH combined with MgSO_4_ treatment

Subsequently, a statistical analysis was performed to identify factors within the ACTH combined with MgSO_4_ group that could potentially influence treatment outcomes. The dependent variable was treatment outcome, while baseline quantitative and qualitative data were considered independent variables, and logistic regression was employed for evaluation. Before PSM, two factors exhibited statistical significance, including the lead time to treatment and the number of ASMs taken before ACTH combined with MgSO_4_ treatment. We found that patients who had taken ≥ 2 types of ASMs before ACTH combined with MgSO_4_ treatment had a 63.4% increased risk of ineffective treatment compared to those with < 2 types (*P* = 0.012, 95% CI = 1.116–2.394). Furthermore, the risk of ineffective treatment increased by 4.3% as the lead time to treatment increased by an additional month (*P* = 0.04, 95% CI = 1.002–1.087). However, after conducting PSM and reperforming logistic regression analysis, only these two factors remained statistically significant. The risk of ineffective treatment increased by 67.9% in the patients who had taken ≥ 2 types of ASMs before ACTH combined with MgSO_4_ treatment compared to those who had taken < 2 types of ASMs (*P* = 0.026, 95% CI = 1.064–2.649). Additionally, the risk of ineffective treatment increased by 5.7% if the lead time to treatment increased by 1 month (*P* = 0.043, 95% CI = 1.002–1.115).

### Subgroup analysis of the lead time to treatment and outcome

**Fig. 2:**
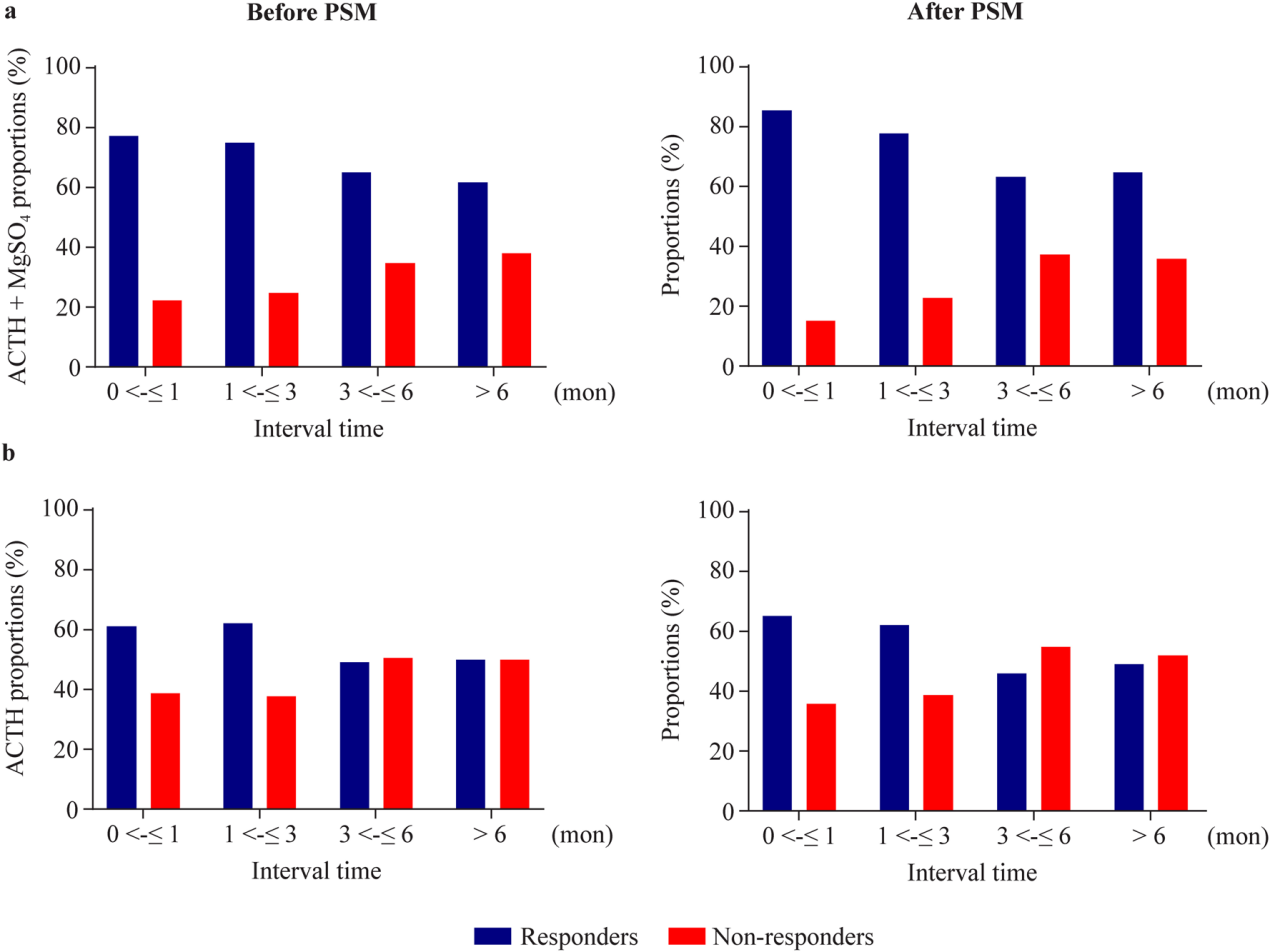
Lead time to treatment and response rate. **a** The response rates of IESS patients in different lead time to treatment groups after PSM who underwent ACTH combined with MgSO_4_ treatment; **b** the response rates of IESS patients in different lead time to treatment groups after PSM who underwent ACTH treatment. *PSM* propensity score matching, *IESS* infantile epileptic spasms syndrome, *ACTH* adrenocorticotropic hormone, *MgSO*_*4*_ magnesium sulfate

Subsequently, subgroup analysis was conducted based on the lead time to treatment and treatment outcomes. Patients were categorized into four subgroups according to their lead time (0 <–≤ 1 month, 1 <–≤ 3 months, 3 <–≤ 6 months,  > 6 months). Following ACTH combined with MgSO_4_ treatment, the response rates of patients in these subgroups were 77.3%, 75%, 65%, and 61.8%, respectively. There was a statistically significant difference in response rates observed across these subgroups (*χ*^2^ = 12.693, *P* = 0.005). Patients with a lead time to treatment of less than 3 months exhibited a significantly better response to ACTH combined with MgSO_4_ treatment than those who had a lead time to treatment exceeding three months (*P* < 0.05). After performing PSM matching, the response rates in the four subgroups treated with ACTH combined with MgSO_4_ were 84.8%, 77.2%, 62.8%, and 64.3%. Significant differences persisted among these groups (*χ*^2^ = 13.539, *P* = 0.004) (Fig. 2a). Similar trends were also evident in the ACTH treatment group (Fig. 2b).

### Multivariate analysis of factors influencing the treatment effect of hypsarrhythmia EEG

**Fig. 3:**
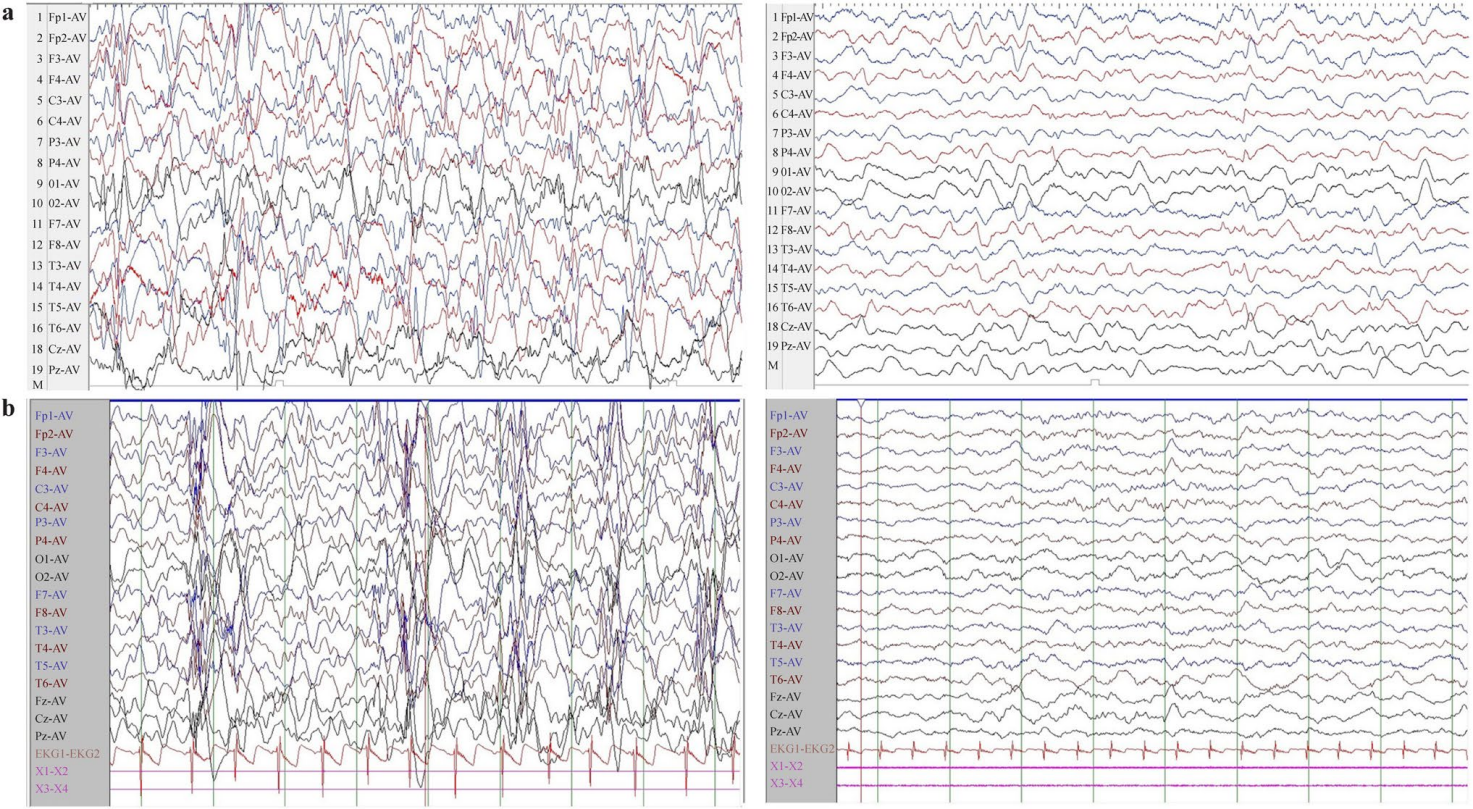
The alterations in hypsarrhythmia EEG before and after treatment of two IESS patients. **a** A female infant aged 6.8 months who was diagnosed with IESS and presented significantly hypsarrhythmia EEG patterns (chaotic, high amplitude, excessive slowing, multifocal epileptiform discharges). After receiving treatment with ACTH combined with MgSO_4_, the hypsarrhythmia EEG was effectively resolved; **b** a male infant aged 7.3 months who was also diagnosed with IESS and exhibited hypsarrhythmia EEG. However, after undergoing a combined treatment protocol, he demonstrated substantial improvement toward normalization. *IESS* infantile epileptic spasms syndrome, *EEG* electroencephalography, *ACTH* adrenocorticotropic hormone, *MgSO*_*4*_ magnesium sulfate

To further investigate additional factors that may influence the resolution rate of hypsarrhythmia EEG, we conducted a logistic regression analysis with the hypsarrhythmia EEG results as the dependent variable and treatment protocol and baseline quantitative and qualitative data as independent variables. Interestingly, following PSM, we found that only the treatment protocol emerged as a significant factor influencing the resolution rate in hypsarrhythmia EEG. After treatment, the risk of children in the ACTH treatment group still exhibiting hypsarrhythmia EEG was 1.502 times higher compared to those in the ACTH combined with MgSO_4_ treatment group (*P* < 0.001, 95% CI = 1.776–3.524). This finding suggests that ACTH combined with MgSO_4_ may have an advantage in improving hypsarrhythmia EEG. The alterations in hypsarrhythmia EEG before and after treatment with ACTH combined with MgSO_4_ in IESS patients are depicted in Fig. 3a and b.

### Analysis of the pathogeny causes

**Fig. 4:**
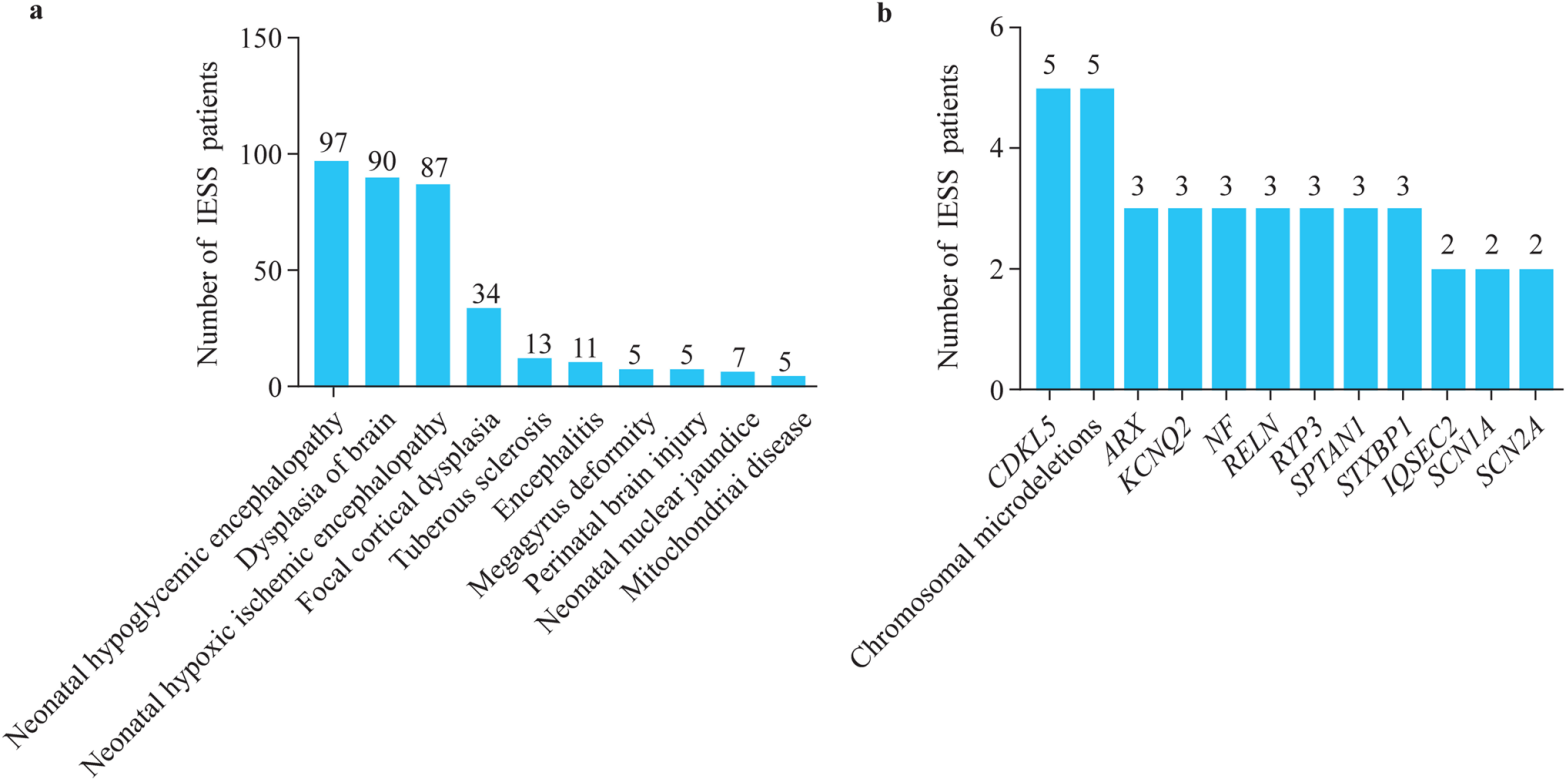
Summary of causes**. a** Top 10 structural/metabolic causes of enrolled IESS patients; **b** number of IESS patients caused by genetic variations. *IESS* infantile epileptic spasms syndrome, *CDKL5* cyclin-dependent kinase like 5, *ARX* aristaless-related homeobox, *KCNQ2* potassium voltage-gated channel subfamily Q member 2, *RELN* Reelin, *RYP3* ryanodine receptor type 3, *SPTAN1* alpha-II-spectrin, *STXBP1* syntaxin-binding protein 1, *IQSEC2* IQ motif and SEC7 domain containing protein 2, *SCN1A* sodium voltage-gated channel alpha subunit 1, *SCN2A* sodium voltage-gated channel alpha subunit 2

We subsequently conducted a statistical analysis of the etiology of the 1005 IESS patients included in the study. Among these IESS patients, 45.57% (458/1005) had identifiable causes. Of these patients with a clear cause, 84.28% (386/458) were attributed to structural/metabolic factors, while the remaining 15.72% (72/458) were due to genetic factors (Table 1). Subgroup analysis of the 386 IESS children attributed to structural/metabolic factors revealed that neonatal hypoglycemic encephalopathy accounted for the highest proportion at 25.13% (97/386), followed by cerebral dysplasia at 23.32% (90/386), neonatal hypoxic-ischemic encephalopathy at 22.54% (87/386), focal cortical dysplasia at 8.81% (34/386), and tuberous sclerosis at 3.37% (13/386) (Fig. 4a). Additionally, among the 72 IESS patients with genetic causes identified, disease-associated gene mutations were detected in 67 cases, and disease-associated chromosomal microdeletions were detected in five cases (Supplementary Table 1). The top ten most frequently observed gene variations are shown in Fig. 4b. Cyclin-dependent kinase-like 5 gene mutation was detected in five cases as the most prevalent alteration. We also observed mutations in genes such as aristaless-related homeobox, potassium voltage-gated channel subfamily Q member 2, NF, Reelin, ryanodine receptor type 3, alpha-II-spectrin, and syntaxin-binding protein 1 among IESS patients.

### Analysis of the incomplete responders

After two weeks of treatment, 71 patients had resolution on hypsarrhythmia EEG but had a reduction rate of seizure frequency less than 50% or even worsened. There were another 152 patients who had no resolution on hypsarrhythmia EEG but had a reduction rate of seizure frequency equal to or greater than 50%. The clinical characteristics of these patients can be seen in Table 3. After PSM, we selected 48 patients from the group with resolved hypsarrhythmia EEG but persistent seizure frequency and 103 patients from the group with unresolved hypsarrhythmia EEG but significantly reduced seizure frequency. In the group with resolved hypsarrhythmia EEG but persistent seizure frequency, the average age of patients was 10.8 months, more boys (64.6%), the average age of onset was 4.8 months, the average lead time to treatment was 6.1 months, an average of 2.2 types of ASMs were taken before receiving treatment, the pathogeny cause of half of them could be characterized, and 72.9% of the patients received the combination treatment. In the group with unresolved hypsarrhythmia EEG but significantly reduced seizure frequency, the average age was 9.9 months, more boys (68.9%), the average age of onset was 4.5 months, the average lead time to treatment was 5.4 months, the pathogeny cause for 35% of the patients could be characterized, and 68% of the patients received the combination treatment. The statistical analysis did not identify any factors that may cause significant differences between the two groups.

**Table 3:** Clinical characteristics of incomplete responders

Variables	All incomplete responders			After PSM		
	(±) (*n* = 71)	(-/+) (*n* = 152)	*P*	(±) (*n* = 48)	(-/+) (*n* = 103)	*P*
Age (mon)	11.4	10.4	0.160	10.8	9.9	0.300
Gender, *n* (%)						0.600
Male	43 (67.6)	93 (61.2)	0.930	31 (64.6)	71 (68.9)	
Female	28 (32.4)	59 (38.8)		17 (35.4)	32 (31.1)	
Age of onset (mon)	5.0	4.7	0.505	4.8	4.5	0.580
Lead time to treatment (mon)	6.4	5.6	0.290	6.1	5.4	0.400
Number of ASMs taken before treatment	2.4	2.4	0.940	2.2	2.2	0.900
Etiological classification, *n* (%)			0.260			0.100
Known	38 (53.5)	71 (46.7)		24 (50.0)	36 (35.0)	
Unknown	33 (46.5)	81 (53.3)		24 (50.0)	67 (65.0)	
Treatment protocol, *n* (%)						0.500
ACTH + MgSO_4_	58 (81.7)	118 (77.6)	0.490	35 (72.9)	70 (68.0)	
ACTH	13 (18.3)	34 (22.4)		13 (27.1)	33 (32.0)	
Other types of epilepsy before diagnosis, *n* (%)			0.610			0.700
Yes	16 (22.5)	39 (25.7)		9 (18.8)	22 (21.4)	
No	55 (77.5)	113 (74.3)		39 (81.3)	81 (78.6)	

*PSM* propensity score matching, *ASMs* antiseizure medications, *ACTH* adrenocorticotropic hormone, *MgSO*_*4*_ magnesium sulfate, *IESS* infantile epileptic spasms syndrome, *EEG* electroencephalography. (±): IESS patients who had resolution on hypsarrhythmia EEG but had a reduction rate of seizure frequency less than 50% or even worsened; (-/+): IESS patients who had not resolution on hypsarrhythmia EEG but had a reduction rate of seizure frequency equal to or greater than 50%

### Safety analysis

Among the 814 IESS patients receiving ACTH combined with MgSO_4_ treatment and 287 IESS patients receiving ACTH alone treatment (including those patients who did not complete the full two weeks of treatment) (Fig. 1), a total of 397 patients (256 patients receiving ACTH combined with MgSO_4_ treatment and 181 patients receiving ACTH treatment) had adverse reactions/events (Table 4). The most common adverse reaction was infection caused by low immunity due to ACTH administration, with 115 cases in the ACTH combined with MgSO_4_ treatment group and 94 cases in the ACTH treatment group (*P* < 0.001). Electrolyte disorders and hypertension were also significantly different between the two groups (Table 4). No statistically significant differences were observed between the two groups in terms of external heart rate, bradycardia, anemia, constipation, intussusception, anal fissure, or abnormal eye movements, which should be considered adverse events. In addition, serious adverse complications, including infection, diarrhea, allergic rash or allergic reaction, irritability and arrhythmia, occurred in 72 patients treated with ACTH combined with MgSO_4_ and 23 patients treated with ACTH alone (Table 4). Among these events, only infection (*P* = 0.045) and hypertension (*P* = 0.025) were significantly different between the two groups, and no deaths occurred during the treatment. Overall, the incidence of adverse reactions/events in the ACTH combined with MgSO_4_ group was 31.4%, which was lower than that in the ACTH group (*P* < 0.001). The total incidence of infection in the ACTH combined with MgSO_4_ group was 21.1%, which was lower than that in the ACTH group (*P* < 0.001). The total incidence of hypertension in the ACTH combined with MgSO_4_ group was 0.5%, which was also lower than that in the ACTH group (*P* < 0.001).

**Table 4:** Number of IESS patients with adverse reactions to the treatment

Variables	ACTH combined with MgSO_4_ (*n* = 814)		ACTH (*n* = 287)		Chi-square test	
	Adverse reactions/events (*n* = 184)	Serious adverse reactions/events (*n* = 72)	Adverse reactions/events (*n* = 158)	Serious adverse reactions/events (*n* = 23)	*P1*^a^	*P2*^b^
Hypertension	3	1	11	4	< 0.001	0.025
Electrolyte disorders	11	0	30	0	< 0.001	–
Hypokalemia	0	2	0	2	–	0.602
Infection	115	57	94	10	< 0.001	0.045
Hypogammaglobulinemia	0	1	0	0	–	1.000
Irritability	8	0	5	0	0.480	–
Allergic rash or allergic reaction	9	3	1	2	0.423	0.841
Diarrhea	17	6	10	1	0.274	0.779
Arrhythmia	5	0	4	1	0.378	0.585
Tachycardia	0	1	0	0	–	1.000
Bradycardia	1	0	2	0	0.344	–
Vomit	1	1	0	2	1.000	0.344
Abnormal liver function	2	0	0	1	0.972	0.585
High muscle tension	1	0	0	0	1.000	–
Abnormal myocardial enzyme spectrum	5	0	0	0	0.412	–
Anemia	2	0	1	0	1.000	–
Constipation	1	0	0	0	1.000	–
Intussusception	1	0	0	0	1.000	–
Anal fissure	1	0	0	0	1.000	–
Abnormal eye movements	1	0	0	0	1.000	–

*IESS* infantile epileptic spasms syndrome, *ACTH* adrenocorticotropic hormone, *MgSO*_*4*_ magnesium sulfate. ^a^Significance of difference for adverse reactions between ACTH combined with MgSO_4_ and ACTH groups; ^b^significance of difference for serious events between ACTH combined with MgSO_4_ and ACTH groups

## Discussion

This study is a large-scale retrospective study based on real-world data. It adopts lenient inclusion and exclusion criteria to cover the widest range of the IESS population. It fits the real clinical environment and has strong feasibility for extrapolating the results. The data of 1005 IESS patients were sourced from the hospital information systems of two authorized medical centers, with good reliability. A strict definition of clinical outcomes combined with EEG, as well as experienced medical and nursing teams, can maximize the avoidance of confounding factors caused by lack of experience or improper care.

The principle of treating IESS patients is to use the minimum amount of medication to achieve the goal of controlling epilepsy with minimal side effects. Hancock and Milner reviewed 18 randomized controlled trials (RCTs) involving 916 patients and 12 treatment drugs, suggesting that single hormone therapy is a better option than vigabatrin for short-term cessation of seizures in IESS [25]. In 2017, an International Collaborative Infantile Spasms Study (ICISS) conducted in the UK found that when steroids (ACTH or prednisolone) were combined with vigabatrin to treat IESS patients, 133 out of 186 (71.5%) enrolled patients experienced complete seizure cessation and disappearance of hypsarrhythmia EEGs after treatment. This response rate was much higher than that of the control group treated with a single type of steroid (56.5%) [26]. This may be attributed to the synergistic effect between ACTH and vigabatrin, as they act on different pathways.

The response rate in our study (68.4%, 95% CI = 65.1%–71.8%) may be slightly lower than that of the ICISS (71.5%). This may be due to more strict inclusion criteria (only IESS patients aged between 2 and 14 months, who have not received any treatment intervention and can initiate treatment within one week after diagnosis, were recruited) and longer treatment time (at least 4 weeks of hormone treatment and continuous 3-month treatment of vigabatrin) in the ICISS study [26]. In the real world, achieving a lead time to treatment of less than one week is challenging. If we used the same criteria of clinical resolution as ICISS, the response rate would be 84.1%. According to the United Kingdom Infantile Spasms Study, the shorter the lead time to treatment is, the better the outcome for IESS patients [27]. Our study also confirmed the relationship between lead time to treatment and clinical outcome (Fig. 2a and b). As IESS is an age-specific epileptic encephalopathy that seriously affects neural development, it is the only epilepsy type that can respond to ACTH treatment. ACTH can promote the synthesis and release of nerve growth factors and accelerate the development of myelin sheaths and dendritic formation in the central nervous system of infants, thereby shortening the possible brain vulnerability period and reducing the damage of epilepsy to the nervous system [28]. Previous studies have shown that the irritable neurohormone corticotropin releasing hormone (CRH) can cause serious seizures and death of neurons in learning- and memory-related areas of the brain, while ACTH may act on CRH neurons located in the hypothalamus through the negative feedback regulation pathway to inhibit the overexcitation of marginal neurons caused by the excessive production and secretion of CRH to achieve anti-epilepsy effects [29, 30]. Both the randomized controlled trial of ICISS and our study found that the response rate of IESS patients to treatment was closely associated with the lead time to treatment [26]. These results indicated that parents and clinical doctors should identify and diagnose IESS as soon as possible and provide the child with the correct treatment for the first time.

In this study, 77.9% of the IESS patients had the resolution of hypsarrhythmia EEG after the combination treatment of ACTH combined with MgSO_4_, which was 18.7% higher than the IESS patients treated with ACTH only (*P* < 0.001) (Table 2). We also found that only the treatment plan was the sole influencing factor for the outcome of high dysrhythmic resolution after multivariate analysis. The benefits of combination therapy in resolving hypsarrhythmia EEG were also observed based on our previous small-scale RCT (73.7% vs. 57.9%, *P* > 0.05), which is consistent with recent RCT study findings (84.6% vs. 46.2%, *P* = 0.04) [21, 31]. Additionally, similar results were obtained from the ICISS study (66.5% vs. 55.0%, *P* = 0.015) [26]. These results indicate that MgSO_4_ is helpful for resolving hypsarrhythmia EEG, which can also enhance the therapeutic effect of ACTH on spastic seizures to a certain extent. MgSO_4_ is a second-line convulsive drug that has been used for over 100 years. In recent years, it has been widely used in the prevention and control of epilepsy, and multiple studies have shown a close relationship between magnesium ions and epilepsy. As a non-competitive antagonist of NMDA, a high concentration of MgSO_4_ in blood can block the NMDA receptor, prevent neuron depolarization, stabilize the cell membrane, and maintain Na^+^/K^+^-ATPase activity during cerebral ischemia [32]. Another possibility is that MgSO_4_ may occupy NMDA channels, preventing calcium ion inflow and the production of free radicals and lipid peroxides, thus producing neuroprotective effects. Furthermore, it cannot be excluded that MgSO_4_ may exert its effects through alternative pathways. Previous investigations have demonstrated that salt-inducible kinase-1 (SIK1), an AMP-activated protein kinase, can interact with Na^+^/K^+^-ATPase and has been proven to play an important role in the ACTH signaling pathway [33, 34]. Recent animal experiments have shown that SIK1 deficiency renders ACTH treatment ineffective in NMDA-induced epileptic spasms. It is worth investigating whether MgSO_4_ exerts its effects on SIK1 through regulation of Na^+^/K^+^-ATPase [35].

Previous animal experiments have shown that low magnesium or magnesium-free perfusate can directly stimulate the hippocampal slices and olfactory bulb slices of rats, leading to spontaneous epileptic discharges [7–9]. Some studies have indicated that epileptic patients have significantly lower levels of magnesium in their plasma and serum compared to control groups [10, 36]. Furthermore, MgSO_4_ infusion has been successfully used to help one drug-resistant epilepsy patient with normal MRI performance and two patients with Alpers syndrome caused by mtRNA polymerase mutations to improve their status epilepticus [12, 13]. In another clinical study involving 22 drug-resistant epilepsy patients, oral magnesium supplementation was found to be a beneficial adjunctive medication for treating drug-intractable epilepsy, as it resulted in a significant decrease in the number of seizure days per month (from 10.2 ± 12.6 days per month to 7.8 ± 10.0 days per month, *P* = 0.004) [14]. A recent study conducted at Boston Children’s Hospital reported that up to 45% of IESS patients died due to sudden and unexpected death caused by uncontrollable status epilepticus [37]. Considering safety, anticonvulsant status and antiepileptic effects, our team chose MgSO_4_ to cooperate with ACTH for the treatment of IESS. In this study, the group receiving ACTH combined with MgSO_4_ demonstrated superior clinical electrical resolution performance, which indirectly confirms that IESS represents a non-convulsive state characterized by persistent epileptic activity.

It is well known that determining the pathogenic cause of a disease is important for selecting a treatment plan. A study on the etiology of 377 IESS patients in the UK found that children with stroke and ischemic hypoxic encephalopathy had better treatment outcomes than those with other causes [38]. This might be related to the protective and preventive effects of MgSO_4_ on brain damage caused by hypoxia and ischemia [39, 40]. We also found that IESS patients with hypoxic-ischemic encephalopathy had a 35% higher response rate to ACTH combined with MgSO_4_ treatment than to ACTH-only treatment. Furthermore, both patients with known or unknown pathogenic causes responded more effectively to combination therapy (Table 2), showing an increase in responders by 14% and 13.7%, respectively. However, this does not mean that determining the pathogenic cause is unimportant. These results inspire us to further investigate the etiology of patients with unknown causes and to apply more advanced diagnostic methods for IESS patients in the future.

We used a low dose level (2–2.5 U/kg/day) of ACTH in this study to avoid adverse reactions. However, we still observed that infection was the most common adverse reaction in the patients, which might be related to the decreased immunity caused by hormone treatment [41]. Nevertheless, most of them returned to normal and subsequently received ACTH or ACTH combined with MgSO_4_ treatment. Although this is the largest real-world retrospective study of IESS conducted thus far, it is not an RCT study, and there are still potential biases despite the use of PSM. This study only investigated the short-term efficacy of the ACTH combined with MgSO_4_ combination regimen and did not investigate the potential long-term outcomes, such as recurrence and neurological development after treatment, which are also crucial for children. In addition, other first-line treatment plans were not set up in this study. Therefore, we cannot report that ACTH combined with MgSO_4_ treatment is the best choice for IESS. Future studies are required to answer these questions.

In conclusion, we analyzed the real-world data of two treatment strategies for IESS patients: ACTH combined with MgSO_4_ and ACTH alone. Our findings support that ACTH combined with MgSO_4_ is a more effective short-term treatment protocol for patients with IESS than ACTH alone, especially for those patients with short lead times to treatment. However, more experiments are required for ACTH combined with MgSO_4_ treatment as a first-line treatment plan for IESS patients.

## Supplementary Information

Below is the link to the electronic supplementary material.

Supplementary file 1 (PDF 52 KB)

## Data Availability

The data of this study are available on reasonable request to the corresponding author.
