# Supplementary material for: Adrenocorticotropic hormone combined with magnesium sulfate therapy for infantile epileptic spasms syndrome: a real-world study
Source: World J Pediatr. 2023 Dec 9;20(8):834–47. doi: 10.1007/s12519-023-00771-1 (PMC11402841; doi:10.1007/s12519-023-00771-1)
Supplement: Supplementary file 2 — Supplementary file 1 (PDF 52 KB) [file 12519_2023_771_MOESM1_ESM.pdf]

Supplementary Table 1. Gene testing results for IESS patients

ACTH combined with $MgSO_4$		ACTH	
Gene	Number of patients	Gene	Number of patients
CDKL5	5	*CDKL5*	1
Chromosomal microdeletions	5	*SCN3A*	1
ARX	3		
KCNQ2	3		
NF	3		
RELN	3		
RYP3	3		
SPTAN1	3		
STXBP1	3		
IQSEC2	2		
SCN1A	2		
SCN2A	2		
ALG13	1		
ASAH1	1		
ATP7A	1		
CACNA1A	1		
CACNA1H	1		
CASR	1		
CDH1	1		
CHD2	1		
CNTNAP2	1		
CUBN	1		
EEF1A2	1		
FLNA	1		
GRIN2B	1		
KCNB1	1		
KCNMA1	1		
MECP2	1		
MMA	1		
MUT	1		
NA1H	1		
NID2	1		
PCDH19	1		
PIGA	1		
RARS2	1		
RBFOX3	1		
SCN3A	1		
SCN8A	1		
SETD5	1		
SLC19A3	1		
SLC25A22	1		

SRPX2	1
STIL	1
TCF4	1
TNK2	1
UBE3A	1
VB6	1

IESS infantile epileptic spasms syndrome, *ACTH* adrenocorticotropic hormone, *$MgSO_4$* magnesium sulfate
